# Local Massage with Topical Analgesic, a Novel Treatment Modality for Temporomandibular Muscular Pain, a Case Study Report of 5 Consecutive Cases

**DOI:** 10.2174/1874325000802010097

**Published:** 2008-05-15

**Authors:** R.W.K Wong, A.B.M Rabie

**Affiliations:** Orthodontics, University of Hong Kong, Prince Philip Dental Hospital, 34 Hospital Road, Sai Ying Pun, Hong Kong

## Abstract

**Introduction:**

Temporomandibular disorders (TMDs) represent a group of painful conditions involving the muscles of mastication and the temporomandibular joint. Ping On Ointment has been used in the Chinese Orthopedics as a soothing massage balm for muscular aches, strain and sprain. If topical application of the ointment can be effective for the treatment of TMD muscular pain, it may be the long-sought-after method for safe, simple, cheap, non-invasive, and effective treatment modality of TMD muscular pain. Purpose: This report documented a case study of the first five consecutive cases using this treatment modality.

**Results:**

All cases resulted in complete remission of pain within one month of topical massage.

**Conclusion:**

This treatment method has high potential to benefit a significant number of people and randomized control trials should be performed.

## INTRODUCTION

Temporomandibular disorders (TMDs) represent a group of painful conditions involving the muscles of mastication and the temporomandibular joint (TMJ) that frequently encountered in general clinical practice, with prevalence in the general population up to 12% [1-3]. Pain can be present at any stage of TMD and is a significant part of the symptoms that prompt patients to seek treatment [4]. Various methods have been used for their treatment; anti-inflammatory or analgesic medications, in particular, are often prescribed in an attempt to decrease the pain associated with TMD. Non-steroidal anti-inflammatory drugs (NSAIDs), as non-selective COX inhibitors (Coxibs), are thought to exert their analgesic and anti-inflammatory effects through both cyclooxygenase-1 (COX-1) and cyclooxygenase-2 (COX-2) inhibition [5, 6]. NSAIDs and coxibs are frequently used for TMD pain [7-9]. Given the prevalence of TMD, safety concerns with chronic NSAID administration, and the controversial nature of non-pharmacologic therapy (TMJ surgery, splints, occlusal alteration) and new treatment modalities for muscular pain related to TMDs are continually being sought.

Chinese Orthopedics is a field of Traditional Chinese that has been used in the Chinese population for the treatment of bone, joint and muscle diseases for several thousands years. One of the methods for treatment of musculoskeletal pain is local massage with topical Chinese medicinal herbs ointment, likes Ping On Ointment. It is possible that this method can be utilized for the treatment of TMD muscular pain.

## BACKGROUND AND SIGNIFICANCE

Ping On Ointment (Ping On Ointment Company Limited, Hong Kong) with ingredients, according to manufacturer, contained Chinese medicinal herbs which include Peppermint oil, 18%; Menthol, 20%; Natural Camphor 6%, Wintergreen Oil, 6%, Sandal-wood Oil, 1%, Eucalyptus Oil, 4%; Bee Wax, 8% and Aromatic Oil, 1%. It does not contain antibiotics, steroid, cortisone or preservatives. It has been used in the Chinese Orthopedics as a soothing massage balm for muscular aches, strain and sprain. It was registered in Hong Kong in 1965 and has been used extensively in China and anecdotal evidence suggests it is effective in reducing painful symptoms. Some of the ingredients (peppermint oil, wintergreen oil and eucalyptus oil) are also used in aromatherapy for pain control. It is the common over-the counter ointment for muscle pain, stomach pain, joint pain and mosquito bite. If topical application of the ointment can be effective for decreasing the severity of TMD muscular pain, it may become one new alternative for simple and non-invasive treatment of TMD muscular pain.

## RESEARCH QUESTION AND AIM

The research question is that topical application of Ping On ointment may be effective for the treatment of TMD muscular pain. The aim of this article is to document a case study of the first five consecutive cases using this treatment modality.

## DESIGN AND METHODS

The basic framework of a case study design was followed [10]. Sample and recruitment of subjects: This was performed on the first five consecutive cases in a university dental clinic which accepted referrals for TMD using this treatment modality for TMD muscular pain.

Human subjects’ protection: Ethical approval has been obtained from the Institutional Review Board of the University of Hong Kong / Hospital Authority Hong Kong West Cluster. All patients were referrals for TMD treatment and consents were obtained. The nature, aim, procedures, possible risks and benefits of the study were explained to the eligible subjects. Both verbal and written informed consents were obtained prior to the screening.

Setting: In all these cases, the diagnosis of TMD was using the research diagnostic criteria for TMDs [11]. They were examined and treated by a qualified dentist with postgraduate training including TMD management. The screening consisted of medical history, questionnaire and clinical examination.

Treatment: These patients were instructed during the clinical sessions to massage the affected area in a circular motion with the ointment for 5 minutes two times daily. The area of application was just on the skin around the temporomandibular joint and affected muscles. The treatment fidelity was determined by regular checking and sending remainders with phone calls.

Dependent variables: The primary dependent variable was the efficacy in the treatment of TMJ and muscle pain as measured by a visual analog scale (VAS, Appendix 2). Subjects needed to complete once daily for up to 4 weeks of application of the ointment. The VAS consisted of a 100 mm line, anchored with the extremes of pain intensity represented as “no pain” and “worst pain possible”. VAS has shown to provide a robust, sensitive, and reproducible method of measuring pain [12]. The patient was asked to give a rating of his pain in the morning, as this was less influenced by other factors occurred during the day. The secondary dependent variable was to assess the mandibular function, the vertical mouth opening, which was the maximal comfortable mandibular opening measured in millimeters at the subject’s maximum incisor to incisor mouth opening using a ruler. This measure of mandibular movement was defined as the maximal inter-incisal distance a subject can open without experiencing evoked pain. This measure has previously been shown to be valid and reliable [13, 14].

All the clinical findings were recorded by the dentist. They were checked for compliance in use of the ointment and they were not using other treatments simultaneously in the treatment of the pain. A summary of the cases is shown in Table **1**.

## CASE 1: MALE, 42 YEARS OLD DENTAL SURGEON

Patient complained very severe TMJ pain on the left side of the face, radiating to the head; there was clicking and muscle spasm on the left side of the TMJ. This condition occurred for less than one month but was not responsive to methyl salicylate ointment treatment. The onset was insidious and the pain duration was whole day. The vertical mouth opening was 20mm with a normal range of 35mm. There was no deviation of the jaw on opening. The left temporalis was severely tender to palpation. Intraoral examination showed a canine guided occlusion and nonworking side interference on the molars on the right side of the dentition.

The patient used Ping On Ointment to massage around the TMJ on the left side. There was an instant improvement of pain and the muscle felt relaxed. The patient then massaged the affected area with the ointment for 5 minutes two times daily. There was improvement in the muscular pain. After two weeks all pain disappeared, the massage was stopped then and there was no recurrence of pain for ten months. The muscle spasm decreased and the vertical mouth opening was 35mm. The clicking was still present on the left side of the joint. The visual analog scale for pain changed from 9 out of 10 to zero before and after treatment.

## CASE 2: FEMALE, 75 YEARS OLD HOUSEWIFE

Patient complained of TMJ pain on both sides of the face together with joint noises. On examination there was crepitus on both sides of the joint. The onset was chronic and insidious and the pain duration was whole day. There was no muscle spasm and the vertical mouth opening was 34mm. There was no deviation of the jaw on opening. Both temporalis muscles were tender to palpation. Intraoral examination showed the molars on the right side were missing and there were no prosthetic replacement for those teeth.

The patient used Ping On Ointment to massage around the TMJ on the both sides with a similar protocol as the above case. There was improvement in the muscular pain. After one week all pain disappeared, the massage was stopped then and there was no recurrence of pain for eleven months. The crepitus was still present on both sides of the joint. The vertical mouth opening was similar before and after treatment. The visual analog scale for pain changed from 7 out of 10 to zero before and after treatment.

## CASE 3: FEMALE, 25 YEARS OLD STUDENT

Patient complained TMJ pain on the left side. She had already received treatment in the University Oral and Maxillofacial TMJ specialist clinic for one year with the pain partially controlled with analgesic. The onset was insidious and the pain duration was whole day. There were occasional limited opening of the mouth and occasional clicking. The vertical mouth opening was 32.5mm. There was no deviation of the jaw on opening. The left temporalis was tender to palpation. Intraoral examination showed the occlusal surfaces of molars were abraded and flat.

The patient used Ping On Ointment to massage around the TMJ on the left side. There was an improvement of pain but it came back when the patient stopped using it after several days. Then patient was informed to continue using that for two weeks. The pain disappeared after one month. After two weeks all pain disappeared and there were no recurrence of pain for nine months. The vertical mouth opening was 35mm. The visual analog scale for pain changed from 7 out of 10 to zero before and after treatment.

## CASE 4: FEMALE, 16 YEARS OLD STUDENT

Patient complained TMJ pain on the left side. Pain occurred when the jaw was moved to the left side or when the jaw was opened maximally. The onset was insidious. There was no muscle tenderness or clicking. The vertical mouth opening was 45mm. There was no deviation of the jaw on opening. The patient was undergoing orthodontic treatment with upper and lower fixed appliances.

The patient used Ping On Ointment to massage around the TMJ on the left side. The pain disappeared after one month. The massage was stopped then and there was no recurrence of pain for six month. The vertical mouth opening was 45mm and there was no pain at maximal opening. The visual analog scale for pain changed from 7 out of 10 to zero before and after treatment.

## CASE 5: FEMALE, 18 YEARS OLD STUDENT

Patient complained TMJ pain on the right side. Pain occurred when the patient was biting hard. The onset was insidious. There was no muscle tenderness or clicking. There was no deviation of the jaw on opening. The patient was undergoing orthodontic treatment with upper fixed appliance only.

The patient used Ping On Ointment to massage around the TMJ on the right side. The pain disappeared after one month. There was no change in the vertical mouth opening. The massage was stopped then and there was no report of pain for seven months. The visual analog scale for pain changed from 8 out of 10 to zero before and after treatment.

## DISCUSSION

The above case reports showed that local massage of topical Chinese analgesic ointment was effective in treatment of TMJ muscle pain. Although TMD is not a single disease entity, it seems that this treatment method is effective in controlling the pain symptom different age groups with different disease stages. It is unlikely that it will treat all the underlying joint and muscle problems as we could see in some cases that the joint sounds still persist. However, there is high hope that this method will be effective in improving the pain symptoms that disturbing a lot of patients for years as we can see it was so effective in treating these patients. The limitation of this study is that it is just a case study. The sample size was small and there was no control groups. Therefore the clinical effectiveness of this ointment on the treatment of TMD could not be scientifically proven based on the data of this study alone. However, this warrants randomized clinical trials to be carried out in a larger population in a more standardized and sophisticated research protocol. The ointment is available online and therefore can be purchased worldwide.

It is possible that other factors e.g. placebo effect, cyclic nature of TMD, might affect the treatment outcome. However, some of the above cases showed cessation of pain for more than half years. And in any cases this ointment was effective in controlling the acute phase of the pain in TMD.

On the other hand, since a lot of other Chinese analgesic ointments are also effective in musculoskeletal problems in Chinese orthopedics, it is possible that massage with other Chinese medicinal analgesic ointments will also be effective in the treatment of the TMD related pain. Further research is needed to demonstrate this.

There is a concern about the toxicity of wintergreen oil overdosage. The wintergreen oil contains a high content of methyl salicylate which may be lethal if high dosage is used, particularly in children [15]. An evidence-based consensus guideline for out-of-hospital management about salicylate poisoning was recently published [16]. Peppermint oil has a long history of use for digestive disorders. It is well tolerated at the commonly recommended dosage but it may cause significant adverse effects at high dosages [17]. An evidence-based consensus guideline for out-of-hospital management about camphor poisoning was published [18]. There was no toxic incidence reported for Ping On Ointment. Given the amount of ointment applied to the TMJ area is small, and the frequency of application recommended is only twice daily. It would be unlikely to have toxic effect if this recommended procedure is followed. However, it is prudent that patients should be warned against frequent application of this and other related ointments in order to avoid side effects of the essential oils and other components like camphor.

Besides, the underlying mechanism needs further investigation. It is postulated that after massage the ointment transmits through the skin, muscles and joint structure and exerts its anti-inflammatory or analgesic effect to the surrounding area. It is possible that the methyl salicylate in the Wintergreen oil, which is one of the NSAIDs mentioned in the introduction, contributes to some of its effects. Peppermint oil has been shown to be effective for relaxing and smoothing muscle and was effective in the treatment of tension and headache, and may have action on the vascular tissue [17]. In addition, study on the analgesic and anti-inflammatory effects of essential oils of Eucalyptus showed that they possess central and peripheral analgesic effects as well as neutrophil-dependent and independent anti-inflammatory activities [19]. Menthol has been used since antiquity for medicinal purposes, but it is only the relatively recent discovery of thermosensitive cation channels in the sensory nerves that finally provides the answer to how menthol can elicit the same cool sensation as low temperatures [20]. Menthol is widely used in dermatologic practice, where it is frequently part of topical antipruritic, analgesic, antiseptic, and cooling formulations. It has an excellent safety as well as toxicity profile, and is currently used as a vehicle in a host of topical and transdermal formulations [20]. Camphor is a naturally occurring compound that is used as a major active ingredient of balms and liniments supplied as topical analgesics. Despite its long history of common medical use, the underlying molecular mechanism of camphor action is not understood. Capsaicin and menthol, two other topically applied agents widely used for similar purposes, are known to excite and desensitize sensory nerves by acting on two members of transient receptor potential channel superfamily. Camphor has also been shown to activate them [21]. Aromatic oil was also shown to have analgesic effect in the treatment of lower back pain [22]. Local massage also increases the local circulation to increase the transmission of the drug. Other questions needed to be asked are: What actually caused the therapeutic effect? Was it the properties of the ointment or the regular effects of the massage when the ointment was being applied? How do you know that the ointment did not just provide an effective lubricant for massage that like has been shown for other forms of manual therapy, perhaps it has a neurophysiological effect on descending pain inhibitory systems [23, 24]? All these are important considerations in future clinical trials testing the ointments effectiveness. Further research is also needed to investigate the effect of the Ping On Ointment on the joint tissue at the biochemical and molecular levels and to identify the combined effects of different active ingredients involved.

## Figures and Tables

**Table 1.** Summary Table for the Cases

	Case 1	Case 2	Case 3	Case 4	Case 5
**Age, Gender**	42, M	75, F	25, F	16, F	18, F
**Presentation of TMD**	Left Side	Both Sides	Left Side	Left Side	Right Side
**Pain description at baseline**	Severe pain Radiating to the head Tender temporalis Muscle spasm Mouth opening 20mm Clicking	Tender temporalis Mouth opening 34mm Crepitus both sides	Pain partially controlled with analgesic Tender temporalis Mouth opening 32.5mm Abraded teeth	Pain increased with mouth opening Mouth opening 45mm Orthodontic treatment	Pain increased with biting Mouth opening 36mm Orthodontic treatment
**VAS before treatment**	9/10	7/10	7/10	7/10	8/10
**Duration of symptoms**	Whole day	Whole day	Whole day	During function	When biting
**Mode of onset**	Insidious	Insidious	Insidious	Insidious	Insidious
**Instructions provided**	Massage with ointment on left TMJ 5 min twice a day	Massage with ointment on both TMJ 5 min twice a day	Massage with ointment on left TMJ 5 min twice a day	Massage with ointment on left TMJ 5 min twice a day	Massage with ointment on right TMJ 5 min twice a day
**Instructions actually carried out**	Continued for 3 weeks	Stopped after 1 week	Stopped after several days Recur, then continued for a further 2 weeks	Stopped after 1 month	Stopped after 1 month
**Pain description after treatment**	All pain disappeared after 2 weeks No recurrence for 10 months Mouth opening 35mm Clicking	All pain disappeared after 1 week No recurrence for 11 months Mouth opening 34mm Crepitus	Pain improved for several days. Recur after application stopped. No recurrence for 9 months after second application Mouth opening 35mm	All pain disappeared after 1 month No recurrence for 6 months Mouth opening 45mm	All pain disappeared after 1 month No recurrence for 7 months Mouth opening 36mm
**VAS after treatment**	0/10	0/10	0/10	0/10	0/10

## APPENDIX 1

The inclusion criteria were:

1. Positive clinical diagnosis of Group I (Muscle disorders) of TMDs. The TMD diagnosis is classified using axis I of the research diagnostic criteria (RDC) for TMDs.
2. For joint and muscle pain complaint, subjects will be required to have a self-report of at least 1 month of daily or nearly-daily pain.
3. 	Subjects with myogenic pain will be included if they meet inclusion and exclusion criteria since patients with TMDs are known to exhibit muscle pain secondary to their joint dysfunction.

The exclusion criteria were:

1. Subjects with infectious arthritis, crystal induced arthropathies, musculoskeletal disorders, subjects with a primary diagnosis of myofascial pain based on the RDC;
2. Subjects with pain attributable to confirmed migraine or head pain condition other than tension headache;
3. Subjects with acute infection or other significant disease of teeth, ears, eyes, nose or throats; subjects with untreated depressive disorder or not on stable antidepressant medicaiton for more than 6 months;
4. Subjects with dental diseases that required ongoing treatment, which would confound the evaluation of orofacial pain;
5. Subjects who are not competent in giving consents.
6. Pregnant or lactating women.
7. Subjects with sensitivity to the ingredient of Ping On Ointment will be excluded.

## APPENDIX 2

Visual Analogue Scale

No Pain  Worst Pain Possible

Instructions for TMD muscle pain Visual Analogue Scale

Mark an “x” on the line which indicates a range of feelings. For instance, if you were feeling “very painful”, you would indicate it on the line below as such:

No Pain  Worst Pain Possible

However, if you were feeling only “mildly to moderately painful” you would indicate it on the line below as such:

No Pain  Worst Pain Possible

The patient to be asked to give a rating of the TMJ pain in the morning after waking up.
